# *Toxoplasma gondii* infection in children with lymphoma in Eastern China: seroprevalence, risk factors and case–control studies

**DOI:** 10.1017/S0950268819001869

**Published:** 2019-11-26

**Authors:** Yuhe Duan, Yunlai Zhi, Yusheng Liu, Na Zhou, Fujiang Li, Xiwei Hao, Xiangyan Zhang, Qian Dong, Lili Chen

**Affiliations:** 1 Department of Pediatrics, The Affiliated Hospital of Qingdao University, 16 Jiangsu Road, Qingdao 266003, China; 2 Department of Urinary Surgery, The People's Hospital of Lianyungang, 182 Guanbei Road, Lianyungang 222002, China; 3 Department of Pathology, The Affiliated Hospital of Qingdao University, 16 Jiangsu Road, Qingdao 266003, China; 4 Department of Nephrology, The Affiliated Hospital of Qingdao University, 16 Jiangsu Road, Qingdao 266003, China

**Keywords:** Children, lymphoma, risk factors, seroprevalence, *Toxoplasma gondii*

## Abstract

Epidemiological data for *Toxoplasma gondii* regarding malignancy have gained increasing attention; however, the information about *T. gondii* infection among children with malignant lymphoma (ML) in China is unclear. Therefore, 314 children with lymphoma and 314 healthy children, age- and gender-matched, were recruited to estimate the seroprevalence of *T. gondii* in the participants and identify the risk factors of infection. Blood samples from all participants were collected and examined for *T. gondii* IgG and IgM antibodies using ELISA. The results showed that the overall seroprevalence of *T. gondii* antibodies (including IgG and/or IgM) in ML patients and healthy controls was 19.8% and 9.9%, respectively. Contact with the cats, consumption of oysters and history of chemotherapy were estimated to be the risk factors for *T. gondii* infection in children with lymphoma by multivariable logistic regression analysis, whereas in healthy children, contact with cats and consumption of oysters were the risk factors. Moreover, among various histological types of lymphoma, individuals with NK/T-cell lymphoma, B-small lymphocytic lymphoma, marginal zone B-lymphoma and Hodgkin's lymphoma had a higher seroprevalence than healthy controls (*P* < 0.05). These findings indicated the high prevalence of *T. gondii* infection in children with lymphoma, and hence, efforts should be performed to evaluate the effect of the infection further in lymphoma patients.

## Introduction

*Toxoplasma gondii* is an apicomplexan parasite that infects approximately one-third of the world population [1]. Humans are infected with *T. gondii* mainly by ingesting cysts from unsanitary food, ingesting food contaminated with cat-derived *T. gondii* oocysts, as well as transmission from mother to the foetus [2].

Efficient immunity can limit the relapse of *T. gondii* infection in the multiplying tachyzoite stage, and hence, acute infection is usually asymptomatic in immunocompetent individuals. However, for the immunocompromised host, the cysts can infect various organs, such as the liver, spleen and nervous system, which, in turn, cause severe symptoms as well as death [3, 4]. Currently, *T. gondii* infection in patients with malignancy is of great concerns, and thus, the correlations between *T. gondii* infection and malignancy have been evaluated [5–7].

Malignant lymphoma (ML) is a common malignancy in children, with more than 13 000 new cases and 1800 disease-related deaths in China in 2015 [8]. The genetic, physical and chemical factors are responsible for the development of lymphoma [9, 10]. Also, a potential correlation between *T. gondii* infection and lymphoma has been reported [11–13]; however, a study conducted by Stamatovic *et al*. did not show any such association [14], thereby rendering it controversial. A few reports have focused on *T. gondii* infection among children with ML in Eastern China, but little is known about the potential risk factors in this group. Thus, the present study was conducted to explore the seropositivity and risk factors associated with *T. gondii* infection in children with ML.

## Methods

### Subjects

Children with ML were followed up and agreed to participate in this study from July 2012 to October 2018. A total of 314 children with primary ML, who presented to the Affiliated Hospital of Qingdao University, were recruited. In addition, 314 healthy children, age-, gender- and residence-matched to the ML patients, were recruited as controls. None of the participants received intravenous immunoglobulin therapy and/or immunotherapy before enrolment. Written informed consent was obtained from all participants/guardians. The study was approved by the Ethics Committee of the Affiliated Hospital of Qingdao University (No. 201311683).

### Sample and data collection

Approximately 2 ml of venous blood was withdrawn from the participants. Blood samples were left at room temperature for 2 h to allow clotting, followed by centrifugation at 3000 rpm for 10 min. The sera were collected in and stored at −80 °C until further analysis.

### Socio-demographic and clinical data

A structured questionnaire was employed to obtain information about the socio-demographic data, including age; gender; residence area; any history of contact with cats, dogs and swine; consumption of raw/undercooked meat, raw vegetables, fruits and oysters; the source of drinking water; and the parents' occupation [15]. Clinical data collected from the medical examination records encompass the infection status of the mother during pregnancy, history of blood transfusion, chemotherapy and the histological type of ML. Participants/guardians did not know the infection status before the data were collected.

### Serological assay

*T. gondii* antibodies (including IgG and IgM) in sera were tested using the commercially available enzyme immunoassay kits (ELISA) (Demeditec Diagnostics GmbH, Germany) according to the manufacturer's instructions. Sera from the ML patients and healthy children were randomly mixed. Positive and negative controls were included in every assay [15].

### Statistical analysis

The results were analysed using the statistical software SPSS 19.0. For the single variable analysis, *χ*^2^chi-square test or Fisher's exact test was used to assess the association between *T. gondii* seroprevalence and various variables. The risk factors associated with *T. gondii* infection were defined by a multivariable backward stepwise logistic regression analysis. Adjusted odds ratio (OR) with 95% confidence interval (CI) were calculated to identify the effect size of risk factors. A *P*-value < 0.05 was considered statistically significant in the multivariate analysis.

## Results

### Socio-demographic and risk factors of ML children with *T. gondii* infection

The overall seroprevalence of *T. gondii* antibodies in ML patients and healthy controls was 62/314 (19.8%) and 31/314 (9.9%) (*P* = 0.001), respectively. A significant difference (*P* = 0.001) was detected while comparing the seroprevalence of *T. gondii* IgG antibodies between children with ML and healthy children, i.e. 60 ML children (19.1%) *vs.* 31 (9.9%) control subjects. Interestingly, we found 13 (4.1%) ML patients and six (1.91%) healthy children positive for IgM antibodies (*P* = 0.103). The baseline data, including socio-demographic and clinical treatment, are shown in Table 1. In ML patients, the seroprevalence of *T. gondii* was higher in 11–14-year-old patients (13/50, 26%) than in those ⩽2-year-old (7/47, 14.89%), although a not statistically significant difference was detected (*P* = 0.18).

**Table 1.** Seroprevalence of *T. gondii* infection in children with lymphoma and control subjects in eastern China

		Children with lymphoma(*N* = 314)			Controls (*N* = 314)			
Variable	Prevalence of *T. gondii* infection				Prevalence of *T. gondii* infection			
	No. tested	No. positive	%	*P*	No. tested	No. positive	%	*P*
Age (years)								
⩽2	47	7	14.9	Reference	50	0	0.0	Reference
3–6	123	21	17.1	0.73	122	14	11.5	0.011^a^
7–10	94	21	22.3	0.29	88	10	11.4	0.014^a^
11–14	50	13	26.0	0.18	54	7	13.0	0.013
Gender								
Male	116	25	21.6	0.54	124	13	10.5	0.77
Female	198	37	18.7		190	18	9.5	
Residence area								
Urban	140	28	20.0	0.92	231	25	10.8	0.35
Rural	174	34	19.5		83	6	7.2	
Contact with cats								
Yes	129	36	27.9	0.002	118	18	15.3	0.01
No	185	26	14.1		196	13	6.6	
Contact with dogs								
Yes	88	16	18.2	0.66	52	5	9.6	0.95
No	226	46	20.3		262	26	9.9	
Contact with swine								
Yes	51	7	13.7	0.24	42	4	9.5	0.98^a^
No	263	55	20.9		272	27	9.9	
Consumption of raw/undercooked meat								
Yes	47	10	21.3	0.78	66	4	6.1	0.24
No	267	52	19.5		248	27	10.9	
Consumption of raw vegetables								
Yes	249	50	20.1	0.77	218	22	10.1	0.84
No	65	12	18.5		96	9	9.4	
Consumption of oysters								
Yes	187	44	23.5	0.04	177	23	13.0	0.04
No	127	18	14.2		137	8	5.8	
Source of drinking water								
Tap	225	47	20.9	0.42	227	23	10.1	0.80^a^
Well + river	89	15	16.9		87	8	9.2	
Parent's occupation								
Farmer	168	34	20.2	0.81	150	18	12.0	0.23^a^
Worker	146	28	19.2		164	13	7.9	
Infection status of mothers during pregnancy								
Yes	16	3	18.8	0.73^a^	44	6	13.6	0.57
No	115	19	16.5		68	12	17.7	
Unknown	183				202			
Blood transfusion history								
Yes	207	40	19.3	0.79				
No	107	22	20.6					
Chemotherapy history								
Yes	216	52	24.1	0.004				
No	98	10	10.2					

a Fisher's exact test was used.

Multivariable analysis revealed that contact with cat (OR 2.5; 95% CI 1.4–4.5; *P* = 0.002), consumption of oysters (OR 1.9; 95% CI 1.1–3.6; *P* = 0.035) and history of chemotherapy (OR 2.2; 95% CI 0.88–4.2; *P* = 0.031) were significantly associated with *T. gondii* infection in ML patients, whereas in healthy controls, contact with cat (OR 2.5; 95% CI 1.2–5.4; *P* = 0.017) and consumption of oysters (OR 2.4; 95% CI 1.0–5.6; *P* = 0.042) were the risk factors for the infection (Table 2). Other variables did not show an association with *T. gondii* infection in the present study.

**Table 2.** Multivariable analysis of children with lymphoma and healthy controls and the association with *T. gondii* infection

Variable^a^	Children with lymphoma			Healthy controls		
	OR^b^	95% CI	*P*	OR^b^	95% CI	*P*
Contact with cats	2.5	1.4–4.5	0.002	2.5	1.2–5.4	0.017
Consumption of oysters	1.9	1.1–3.6	0.035	2.4	1.0–5.6	0.042
History of chemotherapy	2.2	0.9–4.2	0.031			

a Backwards stepwise multivariable analysis.

b Adjusted by age.

### Seropositivity of *T. gondii* in children with ML

Table 3 shows the seroprevalence of different histological type of ML. The maximal seroprevalence of *T. gondii* antibodies was detected in children with NK/T-cell lymphoma (38.46%), followed by B-small lymphocytic lymphoma (30.77%), marginal zone B-lymphoma (25.58%) and diffuse large B-cell lymphoma (21.05%). Compared to the control subjects, patients with NK/T-cell lymphoma, B-small lymphocytic lymphoma, marginal zone B-lymphoma and Hodgkin's lymphoma have significantly higher seroprevalence (all *P* < 0.05).

**Table 3.** Clinical diagnosis and seroprevalence of *T. gondii* in children with lymphoma in eastern China

Clinical diagnosis	No. tested	No. positive	%	OR (95% CI)	*P*
Hodgkin's lymphoma	172	32	18.6	2.1 (1.2–3.6)	0.006
NK/T-cell lymphoma	13	5	38.5	5.7 (1.8–18.8)	0.001
Marginal zone B-lymphoma	43	11	25.6	3.1 (1.4–6.8)	0.003
Mantle cell lymphoma	12	1	8.3	0.8 (0.1–6.6)	0.670
Diffuse large B-cell lymphoma	19	4	21.1	2.4 (0.8–7.8)	0.126
T-cell lymphoma	17	1	5.9	0.6 (0.1–4.5)	0.496
Follicular lymphoma	19	3	158	1.7 (0.5–6.2)	0.304
B-small lymphotic lymphoma	13	4	30.8	4.1 (1.2–13.9)	0.039
Other	6	1	16.7	1.8 (0.2–16.1)	0.470

As compared with 9.9% seroprevalence of *T. gondii* antibodies in controls (31/314).

## Discussion

The genetic, physical and chemical factors are known to be responsible for the development of lymphoma [9, 10]. Although some reports have revealed a possible association between *T. gondii* infection and lymphoma [11–13], the infection status of *T. gondii* in children with lymphoma remains unclear. Therefore, we tested the *T. gondii* antibodies, i.e. IgG and IgM in 314 children with lymphoma and 314 healthy controls to explore the seroprevalence and risk factors associated with *T. gondii* infection in children with ML.

In this study, we found a higher seroprevalence of *T. gondii* IgG antibodies in children with lymphoma as compared to the control subjects (19.1% *vs*. 9.9%, *P* = 0.001), suggesting that the exposure to *T. gondii* is common in children with lymphoma. However, the seroprevalence of *T. gondii* IgM antibodies was not significantly different between children with ML and the controls. Reportedly, *T. gondii* IgG antibodies present later than IgM antibodies in the blood, suggesting a recent infection of *T. gondii* [16]. IgM can persist for several years, and in the presence of a positive IgM result, caution must be exercised since a chronic *T. gondii* infection can be erroneously classified as an acute or false positive; in such cases, IgG avidity tests are crucial [17]. In the current study, two lymphoma patients with IgM antibodies solely were diagnosed with toxoplasmosis according to the clinical features and IgG avidity tests. This result was similar to a study conducted by Zhou *et al*. [15]. Thus, it is necessary to notify the doctors to focus on the significance of *T. gondii* IgM seropositivity in children with malignancy, and patients with solely *T. gondii* IgM antibodies should be tested for IgG avidity to avoid misdiagnosis.

Several studies have demonstrated that the positive *T. gondii* antibodies increased with age in healthy children, but in young patients with malignancy, *T. gondii* is likely to occur [5, 15, 18]. This could be attributed to the fact that younger patients with malignancy may be immunocompromised and are inclined towards the *T. gondii* infection [5, 6]. However, the reasons that younger ML patients were susceptible to *T. gondii* infection have not been well explained, thereby necessitating further investigation.

In the present study, the multivariate logistic analysis showed that contact with cat and consumption of oocysts are associated with the *T. gondii* infection. Interestingly, felines are the only definitive hosts for *T. gondii*, and oocytes can be transmitted via cat faeces and cause toxoplasmosis if the oocyst passed by this method was ingested by humans [1]. Some studies also demonstrated that contact with cats was a risk factor for *T. gondii* infection in patients with malignancy [5, 6]. Moreover, oocysts shed by felines can be washed into the sea by rain, and *T. gondii* oocysts can be maintained in the sea for many years [19]. If these oocysts were ingested by oysters, it might be a potential risk factor for the transmission of *T. gondii*. Qingdao is a coastal city, and oysters are a popular snack for people, and hence, the consumption of oysters might increase the possibility of *T. gondii* infection in patients with malignancy [6]. Therefore, publicizing the information of this risk factor could be conducive to prevent *T. gondii* infection. In addition, recent studies in Shandong province showed that contact with cats and consumption of undercooked oysters were the risk factors for *T. gondii* in oral cancer patients [6], and contact with cats and consumption of undercooked meat could increase the risk of *T. gondii* infection in patients with diabetes mellitus [20]. These similar results indicate the necessity to conduct an epidemiological investigation to identify the risk factors for *T. gondii* infection in different diseases.

Previous studies demonstrated that blood transfusion was a risk factor for *T. gondii* infection in patients with malignancy [6, 15, 21]. In the current study, we found among many clinical variables, the seroprevalence of *T. gondii* was only associated with the history of chemotherapy. Chemotherapy is not only an effective treatment for lymphoma but also suppresses the patients' immune system, which would render them vulnerable to *T. gondii*. Moreover, some studies also demonstrated that chemotherapy augments the infection risk of *T. gondii* [22, 23]. Thus, *T. gondii* infection should be monitored in children with lymphoma; if toxoplasmosis was confirmed, microbiotic antibiotic, such as sulfamethoxazole, should be administered to prevent toxoplasmosis [15].

Among the various histological types of lymphoma, the seroprevalence of *T. gondii* in aggressive lymphomas, such as NK/T-cell lymphoma, B-small lymphocytic lymphoma, marginal zone B-lymphoma and Hodgkin's lymphoma, was significantly higher than that in the controls. However, patients with indolent lymphoma present a lower seroprevalence of *T. gondii* as compared to the controls, which was consistent with the current findings [23]. Some reports showed a potential association between *T. gondii* infection and non-Hodgkin's lymphoma [11], intraocular B-cell lymphoma [12] and B-cell lymphoproliferative disorders [23]. Also, *T. gondii* can dysregulate the immune response pathway and reduce lipid synthesis by downregulating the activity of butyrylcholinesterase [24, 25]. In addition, the infection of *T. gondii* RH strain contributes to the high levels of T helper cell type 1 (Th1) cytokine and a robust inflammatory response and breaks the balance between apoptosis and anti-apoptosis [26, 27], thereby leading to an imbalance in the hosts' gene expression, which would result in carcinogenesis [28].

Nonetheless, aggressive chemotherapy and immunosuppressive therapeutics used for treating the patients suffering from aggressive lymphoma cause deficiency in cell-mediated immunity; therefore, these patients were at risk to *T. gondii* infections and inclined to manifest toxoplasmosis [15]. This phenomenon could partially explain the high seroprevalence of the infection in aggressive lymphoma patients. However, further studies are needed to explore the causes of the difference in the seroprevalence in various histological types of lymphoma.

Nevertheless, the present study has some limitations. First, the limited data did not represent the whole of China. Second, the treatment data of the patients were lacking, and hence, the influence of immunosuppressive management for antibody seroprevalence was uncertain. Third, the donors' sera were not tested, and thus, the influence of the donor-derived antibody was unclear.

## Conclusion

The present study revealed that *T. gondii* infection is prevalent in children with lymphoma, and contact with cats, consumption of raw oysters and history of chemotherapy were independently associated with the risk of infection in this patient group. Thus, the clinicians should be careful with this pathogen infection in patients with lymphoma and efforts should be directed towards evaluating the effect of *T. gondii* in lymphoma patients.

## Supplementary material

For supplementary material accompanying this paper visit https://doi.org/10.1017/S0950268819001869. click here to view supplementary material
