# Supplementary material for: Toxoplasma gondii infection in children with lymphoma in Eastern China: seroprevalence, risk factors and case–control studies
Source: Epidemiol Infect. 2019 Nov 26;147:e305. doi: 10.1017/S0950268819001869 (PMC7003632; doi:10.1017/S0950268819001869)
Supplement: Supplementary file 1 [file S0950268819001869sup001.docx]

Table 4. Single variable analysis for healthy controls in adjusting by age (n = 314).

| Variable | OR^a^ | 95%Cl | P |
| --- | --- | --- | --- |
| Gender |  |  |  |
| Male vs Female | 0.9 | 0.4 - 1.9 | 0.81 |
| Residence area |  |  |  |
| Urban vs Rural | 1.6 | 0.6 - 4.1 | 0.31 |
| Contact with cats |  |  |  |
| Yes vs No | 2.5 | 1.2 - 5.3 | 0.018 |
| Contact with dogs |  |  |  |
| Yes vs No | 1 | 0.4 - 2.7 | 0.98 |
| Contact with swine |  |  |  |
| Yes vs No | 1 | 0.3- 3.1 | 0.97 |
| Consumption of raw/undercooked meat |  |  |  |
| Yes vs No | 0.5 | 0.2 - 1.5 | 0.21 |
| Consumption of raw vegetables |  |  |  |
| Yes vs No | 1.1 | 0.5 - 2.5 | 0.85 |
| Consumption of oysters |  |  |  |
| Yes vs No | 2.3 | 1 - 5.3 | 0.05 |
| Source of drinking water |  |  |  |
| Tap vs Well + river | 0.9 | 0.4 - 2.1 | 0.81 |
| Parent's occupation |  |  |  |
| Farmer vs Worker | 0.7 | 0.3 - 1.4 | 0.29 |
| Infection status of mothers during pregnancy |  |  |  |
| Yes vs No | 0.7 | 0.3 - 2.0 | 0.51 |

^a^Adjusted by age
